# A latent class location-scale regression model with an application to calorie intake data

**DOI:** 10.1007/s10865-025-00613-7

**Published:** 2026-01-13

**Authors:** Xingruo Zhang, Juned Siddique, Bonnie Spring, Donald Hedeker

**Affiliations:** 1 Department of Public Health Sciences, The University of Chicago, Chicago, IL, USA; 2 Department of Preventive Medicine, Northwestern University, Chicago, IL, USA; 3 College of Medicine, Florida State University, Tallahassee, FL, USA

**Keywords:** Subgrouping, Longitudinal data analysis, Intraindividual variability, Eating behaviors

## Abstract

This study introduces an innovative approach for analyzing longitudinal behavioral data with hidden patterns in mean (location) and intraindividual variability (scale) trajectories, using location-scale regressions with latent classes in both the location and scale parts of the model. A full Bayesian approach using Stan is adopted for the estimation of the model parameters. Using simulation studies, we demonstrate that our latent class model yields more precise and informative results, especially regarding the scale, in data exhibiting hidden patterns. Simulation results also show that our model can achieve unbiased parameter estimates as well as a high correct classification rate without over-identifying latent classes in data lacking hidden heterogeneity. Our study equips researchers with a practical tool for subgrouping subjects based on both mean and within-subject variability trajectories of longitudinal outcomes. As an illustration, the latent class model is applied to calorie intake data from a weight loss management study. The integration of latent classes into intraindividual variability trajectories of calorie intake facilitates an understanding of dietary behavior consistency, aiding in personalized weight management interventions.

## Introduction

In longitudinal behavioral studies, the trajectory of within-subject (WS) variability, alongside the mean, is often a critical focus for researchers due to its potential to reveal deeper insights into behavioral dynamics (Bielak et al., 2014; Miquelon & Castonguay, 2017). This heightened interest arises from the need to understand the underlying reasons for more consistent behavior, or conversely, for less consistent behavior in certain contexts. Mixed-effects location scale (MELS) models have been used for modeling such trajectories while accounting for between-subject (BS) heterogeneity in intraindividual variability via random effects (Nordgren et al., 2020; Hedeker et al., 2008). For instance, a recent study found that the WS variability in adolescent smokers’ mood decreased significantly over time (Piasecki et al., 2016). Similarly, using an MELS model, Dunton et al. (2013) observed a reduction in WS variability in physical activity self-efficacy throughout the intervention to increase physical activity.

With richer information collected by longitudinal studies, attention has been drawn to distinguishing homogeneous subgroups within the study population. Subgrouping can serve as a preliminary step for investigating factors influencing intraindividual variability of behaviors in subjects. According to Weller et al. (2020), latent class (LC) analysis helps identify subgroups that may benefit from interventions due to their common attributes. Earlier efforts primarily focused on introducing LCs to the mean, also called location, structure of longitudinal data. Verbeke and Lesaffre (1996), Muthén and Shedden (1999), and Xu (1995) developed mixed-effects models in which the random effects follow a mixture distribution. For longitudinal data, these are often called growth mixture models (GMMs). Alternatively, Nagin and Land (1993) introduced latent class growth analysis (LCGA), that focuses on capturing the heterogeneity between subjects solely through their LC memberships. Computer software, including Mplus (Muthén & Muthén, 2017) and PROC TRAJ in SAS (Jones et al., 2001) have been developed to help researchers apply GMMs and LCGA.

Building on this, Elliott (2007) suggested that the idea of LCs can be extended to WS variability, also called intraindividual variability or scale, of a longitudinal outcome. The approach described by Elliot models the location using a cubic spline regression with distinct residual variances for different LCs. Jiang et al. (2015) integrated LC modeling for both location and scale. They used a joint modeling approach consisting of two components. First, they modeled the longitudinal outcome, incorporating LCs in both the location and scale models. Second, they utilized the results from the LC model to inform the modeling of the binary primary outcome. However, both papers described above focus on the case of constant WS variability over time and assume that no covariates, in particular time, influence the WS variability.

Therefore, this paper proposes a Bayesian location-scale regression with LCs in the trajectories of both mean and intraindividual variability. The model assumes that each subject belongs to one of several subgroups, each characterized by distinct mean (or location) trajectories, and to another set of subgroups, each with unique WS variability (or scale) trajectories. We acknowledge that LC methods impose stronger assumptions. Namely, the homogeneity of location and scale parameters within classes may reduce flexibility compared to subject-level random effects. However, the trade-off enables the identification of discrete latent heterogeneity that could be obscured when continuous distributions of individual differences are assumed. The LC-based structure also facilitates more interpretable subgrouping, which can inform tailored interventions. For example, interventions can be customized to address the distinct needs and behavioral profiles of different LCs, which is an approach less straightforward under continuous random effects frameworks. Moreover, computational complexity is a well-documented challenge for MELS models, particularly when multiple random scale effects are included. In such cases, the proposed LC approach offers a practical alternative.

In Sect. 4, we illustrate how the proposed model can be applied to calorie intake data collected by a weight loss management study. This example demonstrates how LCs in intraindividual variability trajectory contribute to precision care by revealing additional insights into subjects’ consistency in calorie intake. In Sect. 5, the proposed method is validated through simulation studies based on the real-life application. We also demonstrate how the parameters of this model can be estimated using Stan (Stan Development Team, 2022). The accessibility of Stan, with its user-friendly interface, enables researchers with limited experience in Bayesian coding to adopt the proposed method readily. Supporting Information provides comprehensive code templates to facilitate implementation.

### Motivating example

Our methods are motivated by the SMART trial, a longitudinal weight loss management study that utilized mobile health (mHealth) tools (Spring et al., 2024). Four hundred participants were enrolled in the SMART study and randomized to be in either the mHealth-only treatment group or mHealth plus weekly coaching treatment group. Those classified as non-responders during the 2, 4, and 8 weeks follow-ups were re-randomized to add one more mHealth component or both an mHealth component and a traditional weight loss treatment component. Participants in this study were assigned calorie goals based on their baseline weight as part of the weight loss initiatives. Treatment was discontinued at the end of week 12.

We used data on daily reported total calorie intake from the first 12 weeks of the study. We used week as our timing variable and treated it as linear, i.e. week = 0 for week 1 of the study, week = 1 for week 2, etc. Because the focus of this study is on WS variability trajectory, only participants with at least two weeks of observations were kept in our models. A specific week of a participant was included if this participant had at least three observations that week. The final dataset contains 25784 observations from 379 participants, with an average of 68.03 observations per participant. The range of observations per participant in the final dataset is from 9 to 84.

On average, the participants consumed 1299 calories per day, with a standard deviation of 499 calories. The minimum reported value, median reported value, and maximum reported value are 1.8, 1248.6, and 9061.1, respectively. Investigating subgroups based on both mean and intraindividual variability trajectories can shed light on the diverse calorie intake journey of our participants. Furthermore, exploring potential associations between these trajectory subgroups and various participant characteristics and behaviors may offer valuable insights into underlying factors contributing to these individuals’ differences in calorie intake.

## Method

### Location-scale regression with latent classes in mean and intraindividual variability trajectories

The model proposed in this paper is a location-scale regression model that utilizes latent classes to quantify BS heterogeneity in both the location and the scale of the outcome. In the proposed model, trajectories within each LC are assumed homogeneous, as described in the LCGA framework.

The longitudinal outcome $Y_{ij}(i=1,2,\ldots ,N$ subjects; $j=1,\;2,\ldots ,n_{i}$ occasions), when assuming a linear trajectory in its mean, is modeled, conditional on the latent location class of subject i, as follows:

(1) $$\left[Y_{ij}|L_{i}=\ell \right]=\beta_{0\ell }+\beta_{1\ell }t_{ij}+\epsilon_{ij},$$

where $L_{i}=1,\ldots ,L$ follows a Multinomial $\left(\pi_{1}^{\mathrm{location}},\ldots ,\pi_{L}^{\mathrm{location}}\right)$ distribution and indicates the location LC that subject i belongs to. Accordingly, $\beta_{0\ell }$ represents the average response for the *l*th location LC when $t_{ij}=0$, and $\beta_{1\ell }$ represents the average linear response slope over time for the lth location LC.

The observation-level residuals, $\epsilon_{ij}$, are assumed to be normally distributed with a mean of zero. Their variance, also known as intraindividual variability or WS variability, is fitted using a log-linear model as follows:

(2) $$\left[\sigma_{\epsilon_{ij}}^{2}|S_{i}=s\right]=exp\left(\tau_{0s}+\tau_{1s}t_{ij}\right),$$

where $S_{i}=1,\ldots ,S$ also follows a Multinomial $\left(\pi_{1}^{\mathrm{scale}},\ldots ,\pi_{S}^{\mathrm{scale}}\right)$ distribution and indicates the scale class that subject i belongs to. $\tau_{0s}$ is the average log WS variance for the sth scale LC at baseline, and $\tau_{1s}$ represents the average trend in intraindividual variability on the log scale for the sth scale LC. In this proposed model, latent classes are specified separately for location and scale to flexibly capture heterogeneity in each component. This structure accommodates cases where individuals share similar location trajectories but differ in variability, or vice versa, and avoids the parameter explosion that would result from modeling all possible location-scale combinations jointly. As a result, we feel that the model remains both parsimonious and interpretable.

Denote observations from subject i as $Y_{i}$. Assuming a priori independence between location LCs and scale LCs, we integrate over the likelihood of $Y_{i}$ conditional on subject i’s location LC and scale LC memberships to obtain their marginal likelihood:

(3) $$L\left(Y_{i}\right)=\Sigma_{\ell =1}^{L}\Sigma_{s=1}^{S}\pi_{\ell }^{\mathrm{location}}\pi_{s}^{\mathrm{scale}}L\left(Y_{i}|L_{i}=\ell ,S_{i}=s\right),$$

where the conditional likelihood is based on that $Y_{ij}|L_{i}=\ell ,S_{i}=s$ follows the normal distribution with a mean of $\beta_{0\ell }+\beta_{1\ell }t_{ij}$ and a variance of $exp\left(\tau_{0s}+\tau_{1s}t_{ij}\right)$. The overall marginal log-likelihood for the study population is obtained by summing the marginal log-likelihoods of all subjects together. Posterior LC membership probabilities for subject i can be derived following Bayes’ theorem.


(4) $$\eta_{i,\ell }^{\mathrm{location}}=P\left(L_{i}=\ell |Y_{i}\right)\;\;=\frac{\pi_{\ell }^{\mathrm{location}}\sum_{s=1}^{S}\pi_{s}^{\mathrm{scale}}L\left(Y_{i}|L_{i}\;=\;\ell ,\;S_{i}\;=\;s\right)}{L\left(Y_{i}\right)};$$

(5) $$\eta_{i,s}^{\mathrm{scale}}=P\left(S_{i}=s|Y_{i}\right)\;\;=\frac{\pi_{s}^{\mathrm{scale}}\Sigma_{\ell =1}^{L}\pi_{\ell }^{\mathrm{location}}L\left(Y_{i}|L_{i}\;=\;\ell ,\;S_{i}\;=\;s\right)}{L\left(Y_{i}\right)}.$$

Classification of each subject is then determined based on the class with the highest posterior mean probability. This can be expressed as ${\tilde{L}}_{i}={argmax}_{\ell }{\overset{ˆ}{\eta }}_{i,\ell }^{\mathrm{location}}$, and ${\tilde{S}}_{i}={argmax}_{s}{\overset{ˆ}{\eta }}_{i,s}^{\mathrm{scale}}$, where ${\overset{ˆ}{\eta }}_{i,\ell }^{\mathrm{location}}$ and ${\overset{ˆ}{\eta }}_{i,s}^{\mathrm{scale}}$ are the posterior means of $\eta_{i,\ell }^{\mathrm{location}}$ and $\eta_{i,s}^{\mathrm{scale}}$ described in Equations 4 and 5.

### Bayesian inference with Stan

Models described in this paper were estimated using a Hamiltonian Monte Carlo (HMC) algorithm implemented by the R interface to Stan (Stan Development Team, 2022). The Stan programs in the Supporting Information are portable to Stan interfaces in multiple programming languages. We chose Bayesian inference over maximum likelihood estimation to avoid heavy calculation of the first- and second-order partial derivatives (McCulloch, 2003). Also, according to Lee and Song (2004), Bayesian inference is more suitable for smaller sample sizes than maximum likelihood estimation and hence can be applied to studies of a wider range of sizes.

Default flat priors were used for all parameters. To temper the potential problem of label switching (Redner & Walker, 1984), we adopted the suggestion from the user manual of Stan (Stan Development Team, 2022), that is, first to run one chain of each model and use the posterior means of parameters from this step as starting values for the model with four chains. The starting values for the first chain were derived from separate regressions for the mean and WS variability of the outcome. Subsequently, the four MCMC chains were run for 2000 iterations, with the first 500 as warm-up iterations. We determined model convergence through trace plots and R-hat convergence diagnostics (Vehtari et al., 2021).

### Choice of numbers of latent classes

Leave-one-out cross-validation (LOO) is a highly recommended method for assessing Bayesian models. In contrast to the Akaike information criterion (AIC) and the deviance information criterion (DIC), LOO offers several advantages: it fully embraces Bayesian principles by utilizing the entire posterior distribution, remains unaffected by parameterization choices, and is applicable even in cases involving singular models. LOO is also favored over the widely applicable information criterion (WAIC) due to its robustness in scenarios with weak priors and/or influential observations and its capability to offer approximate standard errors (SEs) (Vehtari et al., 2017). Furthermore, because marginal likelihoods are incorporated into our model estimation, the use of LOO for class enumeration is particularly well-supported. Tong et al. (2021) demonstrated that LOO performs reliably for selecting the number of latent classes when marginal likelihoods are utilized.

In this study, the *loo* package in R (Vehtari et al., 2017) was applied to calculate LOO expected log pointwise predictive density (elpd) values based on the fitted Stan models. The package uses Pareto-smoothed importance sampling (PSIS) to compute elpd in an efficient, accurate, and stable manner. A higher $elpd_{LOO}$ value is better while accounting for model complexity.

### Application to weight loss management study example

We now revisit the calorie intake data described in Sect. 2 to demonstrate how the proposed method can uncover subgroups with different trajectories in their mean and WS variability. The outcome, total calorie intake, was log-transformed to reduce skewness and improve estimation performance. With sample size and interoperability in mind, we analyzed the dataset with the proposed model with up to three location LCs and three scale LCs. We observed that a larger number of classes leads to increased data sparsity in the classification results. Moreover, models with more than three scale LCs also resulted in Pareto k diagnostic values in the problematic range of above 0.7 (Paananen et al., 2021), indicating unreliable LOO estimates. This suggests the added complexity reduces confidence in the model’s predictive performance. Inspection of the trace plots showed reasonable convergence for all nine models with up to three location LCs and three scale LCs. The $elpd_{LOO}$ values and SEs of all nine models are listed in Table 1. We selected the model with three location and three scale latent classes, as it was the last to show an improvement in model fit measured by a LOO difference of at least four SEs. In other words, the location part of the final model can be expressed as

(6) $$\left[log\left({\mathrm{total\_calories}}_{ij}\right)|L_{i}=\ell \right]=\beta_{0\ell }+\beta_{1\ell }{\mathrm{week}}_{ij}+\epsilon_{ij},\ell =1,2,3.$$

And conditional on participant i’s latent variability trajectory class $S_{i}$, the scale model for participant i is as follows:

(7) $$\left[\sigma_{\epsilon_{ij}}^{2}|S_{i}=s\right]=exp\left(\tau_{0s}+\tau_{1s}{\mathrm{week}}_{ij}\right),s=1,2,3$$

The numbers of participants classified into each of the nine combinations of location LCs and scale LCs are listed in Table 2. The procedure for classification is described in the last paragraph of Sect. 3.1. As recommened by Van Lissa et al. (2024), we examined the entropy values of the selected model. The entropy values are high, namely 0.85 for location classification and 0.88 for scale classification, calculated using the following formulas (Clark & Muthén, 2009). These values close to one indicate good class separation.

(8) $$E_{\mathrm{location}}=1-\frac{\Sigma_{i}\Sigma_{\ell }(-{\overset{ˆ}{\eta }}_{i,\ell }^{\mathrm{location}}\;log({\overset{ˆ}{\eta }}_{i,\ell }^{\mathrm{location}}))}{n\;log(L)};$$

(9) $$E_{\mathrm{scale}}=1-\frac{\Sigma_{i}\Sigma_{s}(-{\overset{ˆ}{\eta }}_{i,s}^{\mathrm{scale}}\;\log\left({\overset{ˆ}{\eta }}_{i,s}^{\mathrm{scale}}\right))}{n\;\log\left(S\right)},$$

where $E_{\mathrm{location}}$ represents the entropy of the location part of the model, and $E_{\mathrm{scale}}$ represents the entropy of the scale part.

**Fig. 1:**
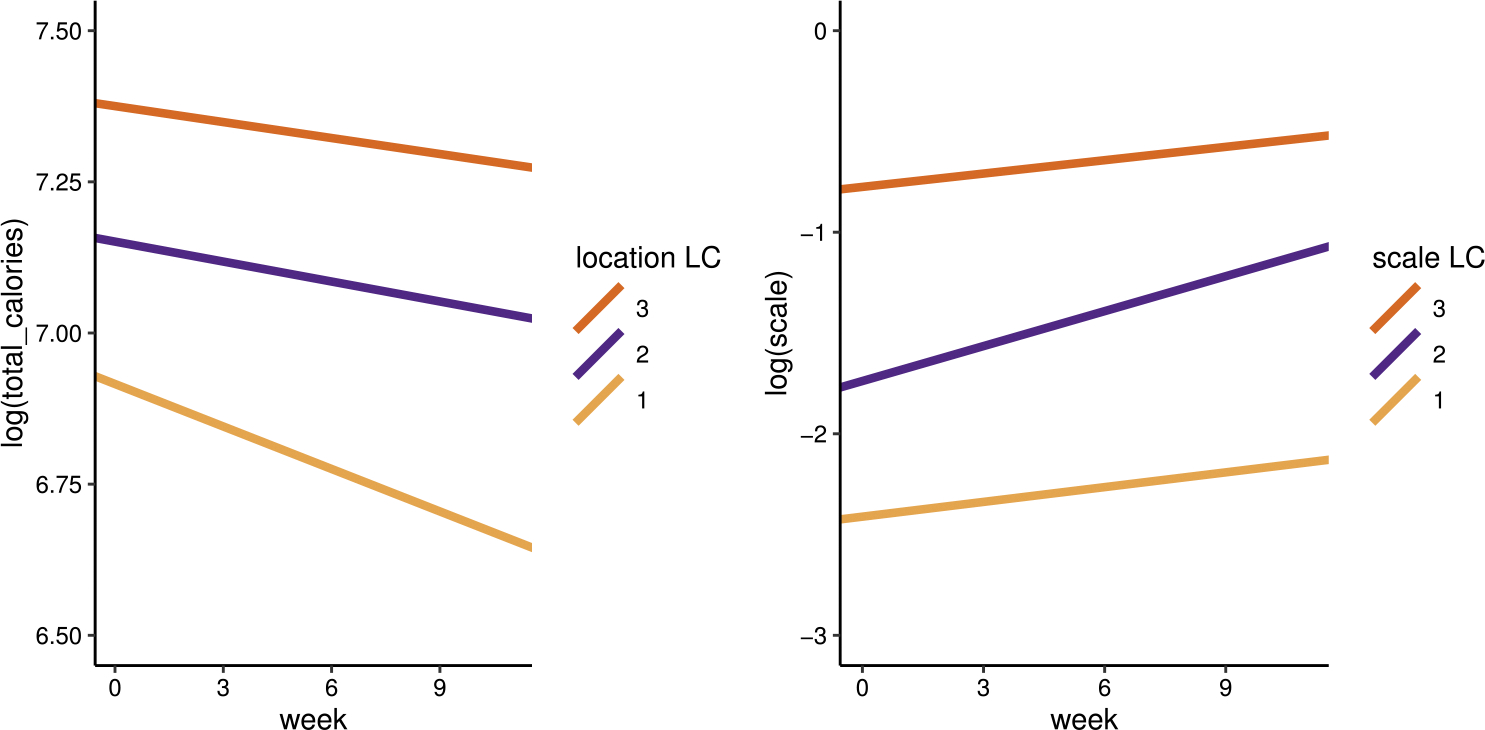
Estimated location and scale trajectories for each LC

The estimated parameters and their 95% credible intervals for the final model described above can be found in Table 3. Visual representations of the location and scale trajectories are presented in Fig. 1. Note that the numbers are small for the slopes due to log-normal modeling; that is, these numbers represent multiplicative trends. The location slopes of −0.023, −0.011, −0.009 correspond to approximately 22.4%, 11.4%, and 9.4% decrease in average calorie intake from baseline to week 12, respectively. Recent research suggests that 20% calorie reduction is associated with health benefits (Picca et al., 2017). Hence, the magnitude of reduction observed in our analysis, especially the largest, aligns with evidence-based targets and supports clinical meaningfulness of these effects.

All three location classes exhibit decreases in calorie intake over time. The first LC started with lower calorie intake on average ($\beta_{0}|L_{i}=1=6.916$) and decreased more compared to the other two LCs. The slopes of mean calorie intake are approximately parallel for location LCs 2 and 3, while the expected log(total calories) at baseline is 7.15 for the 2nd location LC and 7.38 for the 3rd location LC. These findings imply that it might be easier to reduce calorie intake when it initially starts at a lower level, highlighting the potential significance of cultivating healthy eating habits from the outset. For all three scale LCs, calorie intake for each participant also tends to become more variable over time, with the slope of WS variability being more positive for the 2nd scale LC. Thus, for participants in scale LC 2, interventions can potentially prioritize enhancing the consistency in calorie intake. The average WS variability for scale LC 2 at baseline ($exp\left(\tau_{0}|S_{i}=2\right)$), which equals 0.176, is between scale LC 1 ($exp\left(\tau_{0}|S_{i}=1\right)=0.090$) and scale LC 3 ($exp\left(\tau_{0}|S_{i}=3\right)=0.461$).

The most prevalent combination is location LC 2 and scale LC 2, which is the class that started off with medium mean and variability and increased a lot in calorie intake variability over time. A limited number of participants are categorized within the groups denoted by location LC 1 and scale LC 1, as well as location LC 3 and scale LC 3. This observation suggests that within the study population, it is relatively infrequent for individuals to (1) initially exhibit low calorie intake with low WS variability while simultaneously experiencing a significant decrease in calorie intake over time; (2) commence with high calorie intake with high WS variability. These findings might be signs that high calorie intake is a relatively stable behavior and that calorie intake is resistant to change.

**Fig. 2:**
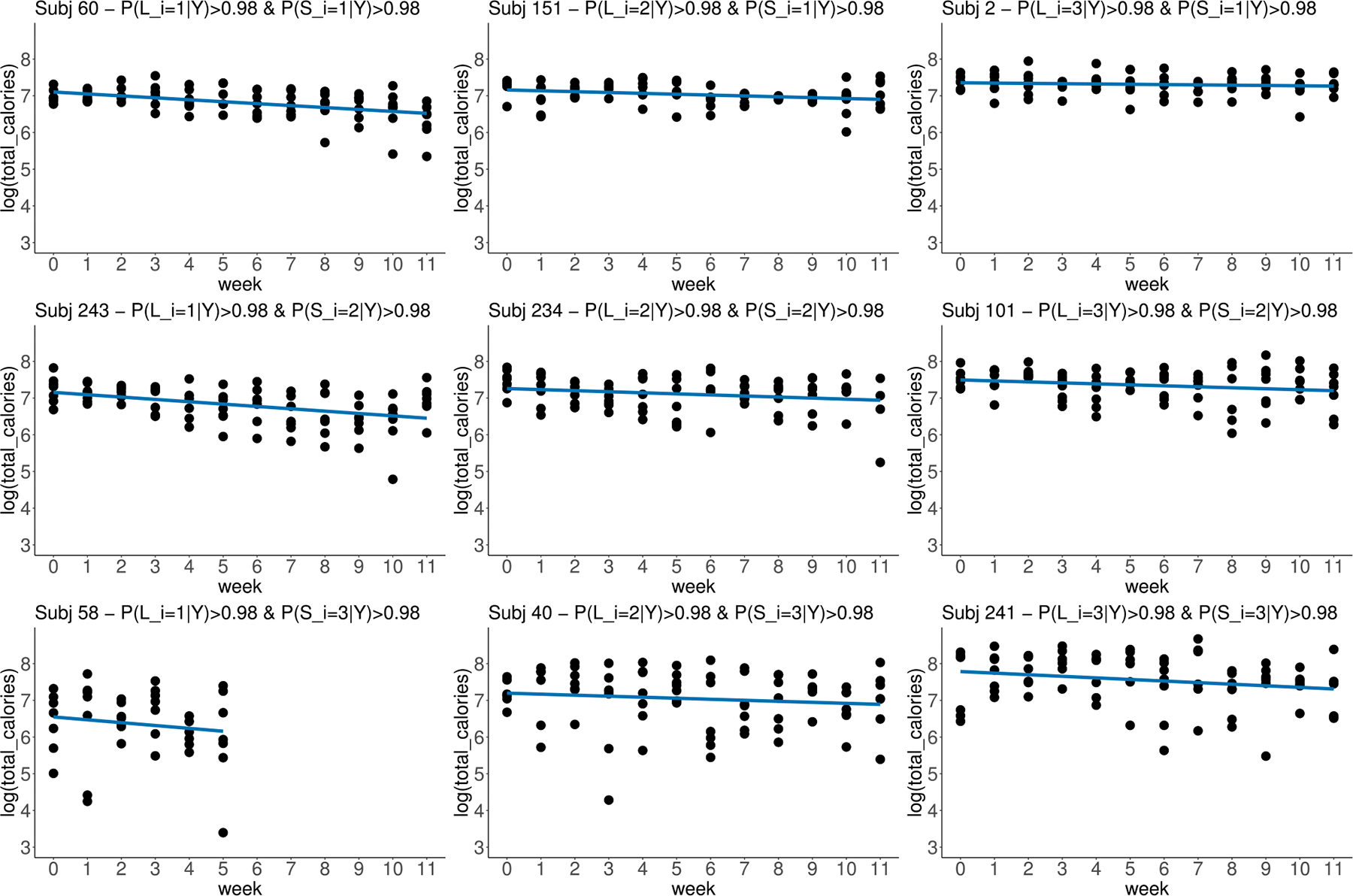
Subjects with high posterior probability of belonging to each LC combination. Blue lines represent trajectories of mean log(total calories) from subject-specific linear regressions

Figure 2 presents data from participants with high posterior probabilities of belonging to each LC combination. Consistent with the parameter estimates, participants 60, 243, and 58, whose posterior probabilities of belonging to location LC 1 are high, exhibited low mean calorie intake at baseline and a more negative trend over time in terms of mean calorie intake; on the contrary, participants 2, 101, and 241, with high posterior probabilities of belonging to location LC 3, started off with high mean calorie intake that did not decrease as much. From top to bottom of Fig. 1, participants belonging to scale LCs 1, 2, and 3 displayed an upward pattern in terms of baseline WS variability in calorie intake. It is also obvious from the plots that the WS variability in calorie intake increased for participants 243, 234, and 101, who belong to scale LC 2.

Sociodemographic characteristics, baseline weight and height, as well as original treatment assignment of the participants, are summarized with regards to their LC classification in Tables 4 and 5. For location—there are more males and a lower percentage of Black or African American participants in location LC 3 compared to the other two location LCs. Both mean weight and mean height at baseline were the lowest for participants classified into location LC 1, higher for those in location LC 2, and highest for those in location LC 3. Scale latent class membership varied with participant characteristics including age at screening, baseline weight, and education level. Participants in scale LC 1 were on average older and lighter at baseline. Participants in scale LC 3 had lower education levels on average at baseline.

### Simulation study

In this section, we use simulated data to evaluate the validity of our method and estimation procedure. Because of the computationally expensive nature of the model estimation process, the simulation study was limited to 100 datasets per scenario.

The simulation study serves two main purposes. First, we aim to demonstrate the validity of the proposed method and highlight issues that arise when the model is under-parametrized. To this end, in Scenario 1, we generated simulated datasets that resemble the SMART study to assess the performance of our proposed model. Second, we seek to show that the proposed method does not over-identify LCs by fitting models with more LCs than are present in the data. Accordingly, in Scenario 2, we simulated datasets that assume all subjects have the same intercept and slope in both the location model and the scale model. In Scenario 3, we simulated datasets with two location LCs and two scale LCs. Therefore, scenarios 2 and 3 represent the over-parameterized cases in our exploration of model misspecification.

In the first scenario, each simulated dataset includes 250 subjects, with each subject being randomly assigned to one of three latent location classes and one of three latent scale classes, each selection having an equal probability. For every subject, we considered data across 12 time periods with ten observations in each time period. The true values of the generating parameters are shown in Table 6.

These datasets were analyzed using (1) location-scale regressions with three location LCs and three scale LCs, (2) location-scale regressions with three location LCs and one scale LC, and (3) regressions incorporating three location LCs without incorporating a scale component. For easy comparison, Table 6 presents the averages of three key metrics for these models: the bias relative to true values, the average width of their corresponding 95% credible intervals, and the coverage rate of the true values. Bias was computed as the average difference between the posterior means and the true parameter values across the simulated datasets. Average interval width (AIW) was defined as the average width of the 95% credible intervals across replications. Coverage was calculated as the proportion of simulations in which the 95% credible interval for a parameter contained the true value.

No label switching was detected for models (1) and (2), while model (1) failed to converge for eight datasets. Label switching happened for four out of 100 datasets for model (3). Therefore, results presented in Table 6 are based on 92 datasets for model (1) and 96 datasets for model (3). The first thing to notice in the simulation results are the small biases of the parameter estimates compared to their true values for Model (1), together with coverage rates around 95%, which suggest that the proposed method is suitable for data like those from the weight loss management study example. The AIWs are wider for all parameters in both Model (2) and Model (3) compared to Model (1).

Simulation results for Model (2) indicate that excluding latent classes (LCs) when modeling intraindividual variability can lead to unreliable scale parameter inferences. Specifically, we compared posterior means and corresponding 95% credible intervals for parameters $\tau_{0}$ and $\tau_{1}$ in Model (2) with true values of each of the three scale LCs. These 95% credible intervals fail to encompass the true values for any of the three LCs. The average estimated value for $\tau_{0}$ is −1.43, while for $\tau_{1}$, it is 0.03, both approximating the mean across the three LCs. This suggests that the model neglects the heterogeneity in initial values and the subsequent variability trends by not incorporating LCs in its scale component. The relatively narrow AIWs observed also suggest that the model fails to capture the full extent of heterogeneity in WS variability. Relating this back to the motivating example in Sect. 4, omitting latent scale classes may obscure important differences in WS variability of exercise behavior among individuals, thereby reducing the potential to personalize future treatment strategies based on these differences.

The average correct classification rates for the location component are notably high across all three models, at approximately 98.6%. Specifically, in Model (1), an average of 96.0% of subjects attain posterior classification probabilities exceeding 90% for a particular location LC. Also for Model (1), a similar high performance is observed in its scale part: 99.8% of subjects are correctly classified on average across all datasets, with an average proportion of 99.6% of subjects achieving posterior classification probabilities of at least 90% for a specific scale LC. These results suggest that the proposed model is highly effective in accurately classifying nearly all subjects with respect to both their location and scale trajectories.

Each simulated dataset in scenarios 2 and 3 also includes 250 subjects, with 12 time periods and ten observations in each time period for every subject. We then analyzed these datasets with the proposed location-scale regression model with one more LC in either the location or scale part of the model or both. We found that these models would not converge, as shown by the trace plots in the first section of the supporting information. Specifically, when the model specifies one more location LC than in the dataset, the chains do not stabilize around a certain value but instead show a visible pattern for one set of β’s. The chains also diverge from each other over iterations instead of converging to a common value. When there is one more scale LC in the model than in the dataset, the fluctuation is substantially wide for a pair of $\tau_{0}$ and $\tau_{1}$. Both findings occur when there are both an additional location LC and an additional scale LC in the model. These results provide compelling evidence that the LCs identified in Sect. 4 are a manifestation of actual heterogeneity in the data, rather than artifacts resulting from modeling limitations. The model convergence failures align with practical challenges when applying complex models to simpler data structures.

## Discussion

In our investigation, we explore the application of location-scale regressions incorporating LCs to model the trajectories of both mean and intraindividual variability of eating behaviors. Through simulation studies, we substantiate the necessity of employing LC-based models when analyzing data exhibiting LC structures. This approach yields more precise parameter estimates and enhances the extraction of valuable insights from complex datasets characterized by LCs. Consequently, our research contributes a practical, user-friendly tool to researchers interested in subject subgrouping based on location-scale trajectory patterns. This innovation will benefit researchers seeking to incorporate intraindividual variability trajectories, either alone or in conjunction with mean trajectories, into the design and evaluation of interventions. Instead of time trajectory, the proposed model can be used to conduct subgrouping in terms of the fixed effects of various time-varying covariates on mean and WS variability. In order to do so, researchers can simply replace the time covariate in the model with the specific time-varying covariate of relevance. In randomized controlled trials, baseline equivalence might be established, and analysis will focus on changes during the course of the trials. Our proposed model can be easily adjusted to assume homogeneous starting points, or intercepts, and have the LCs determined by slopes only.

In the weight loss management study example, our proposed model divided the participants into three subgroups with different trajectories in location and another three subgroups with different trajectories in scale. The selected number of classes is appropriate for our sample size and supported by both the $elpd_{LOO}$ comparison and entropy values. Notably, prior research has substantiated the positive impact of dietary consistency on weight loss outcomes (Rosenbaum et al., 2016). Consequently, there exists an opportunity for in-depth investigation within the subgroup displaying a more noticeable increasing trend in dietary variability. Such an inquiry aims to elucidate the underlying factors driving this transition, with the potential to inform strategies that can be employed to promote dietary consistency within this subgroup. However, it is important to acknowledge that calorie intake in this study relies on self-reporting. For instance, we see some really low daily reported total calorie intake values in Fig. 2. Under-reporting dietary intake in weight loss management initiatives is a known issue (Macdiarmid & Blundell, 1998; Connor, 2020). Further research may be necessary to determine whether the observed variability in calorie intake reflects actual fluctuations or inconsistencies in reporting.

While this paper focuses on linear trajectories in both location and scale, more complicated modeling of either mean and/or WS variability of outcome is possible. Yet, it is essential for readers to consider that these extensions may entail increased computational demands and a higher likelihood of convergence issues. Also, more observations from each subject might be required to achieve meaningful parameter estimates. Also, it is worth noting an alternative modeling approach, namely mixed location scale hidden Markov models (Lin et al., 2020), that emphasizes the sequential relationship of scale. In such sequential scale models, the focus is on time serial dependence of intraindividual variability over time, offering a distinct perspective on within-subject variability. Another assumption made in this study is the a priori independence between location LCs and scale LCs. A potential extension of this study could explore the possibility of incorporating correlated LCs and investigate the implications of such correlations. We also acknowledge that simulation studies examining model performance across a broader range of latent class numbers are of interest, which may become more achievable with future improvements in the computational efficiency of the estimation procedure.

The next steps of this study also include expanding the proposed model to include covariate(s) for the LC membership probabilities and building a joint model in which the primary outcome of a study, for example, weight loss in our example study, can be further modeled as a function of the LCs of subject behaviors. By integrating these components, the model can function as an analytical pipeline, examining the association between factors like treatment assignment and subjects’ LC membership in behavior, followed by investigating the connection between the LC and the study’s outcomes.

## Supplementary Material

supp_1_pdf

**Supplementary Information** The online version contains supplementary material available at https://doi.org/10.1007/s10865-025-00613-7.

## Figures and Tables

**Table 1:** *elpd_LOO_* (SE) for location-scale regressions with different numbers of LCs

Number of LCs in the location model					
Number of LCs in the scale model	1	Δ	2	Δ	3

1	−21338.0 (426.4)	1111.9 (102.7)	−20226.1 (412.5)	379.0 (57.1)	−19847.0 (405.0)
Δ	1404.6 (131.9)		1365.5 (119.4)		1255.6 (116.2)
2	−19933.4 (424.7)	1072.8 (98.9)	−18860.6 (406.8)	269.1 (50.0)	−18591.5 (403.9)
Δ	338.5 (54.5)		326.4 (51.5)		343.0 (51.9)
3	−19594.9 (431.3)	1060.7 (96.9)	−18534.2 (411.4)	285.8 (59.5)	−18248.4 (410.0)

Cells labeled Δ represent pairwise *elpd_LOO_* differences between adjacent models with SEs

**Table 2:** Distribution of subjects among LC combinations

Scale LC	Location LC			Total
	1 [low intercept, most negative slope]	2 [mid intercept, mid slope]	3 [high intercept, least negative slope]	

1 [low intercept, mid slope]	3 (0.8%)	51 (13.5%)	24 (6.3%)	78 (20.6%)
2 [mid intercept, high slope]	65 (17.2%)	97 (25.6%)	38 (10.0%)	200 (52.8%)
3 [high intercept, low slope]	64 (16.9%)	31 (8.2%)	6 (1.6%)	101 (26.6%)
Total	132 (34.8%)	179 (47.2%)	68 (17.9%)	

**Table 3:** Parameter estimates and credible intervals for the application to SMART weight loss management study

Location part of the model				
Location LC	Parameter	Estimate	Parameter	Estimate
		(95% credible interval)		(95% credible interval)

1 [low intercept, most negative slope]	$\beta_{0}|L_{i}=1$	6.916	$\beta_{1}|L_{i}=1$	−0.023
		(6.891, 6.940)		(−0.028, −0.019)
2 [mid intercept, mid slope]	$\beta_{0}|L_{i}=2$	7.151	$\beta_{1}|L_{i}=2$	−0.011
		(7.132, 7.171)		(−0.013, −0.009)
3 [high intercept, least negative slope]	$\beta_{0}|L_{i}=3$	7.375	$\beta_{1}|L_{i}=3$	−0.009
		(7.347, 7.413)		(−0.012, −0.005)
Scale part of the model				
Scale LC	Parameter	Estimate	Parameter	Estimate
		(95% credible interval)		(95% credible interval)

1 [low intercept, mid slope]	$\tau_{0}|S_{i}=1$	−2.411	$\tau_{1}|S_{i}=1$	0.024
		(−2.492, −2.326)		(0.013, 0.036)
2 [mid intercept, high slope]	$\tau_{0}|S_{i}=2$	−1.738	$\tau_{1}|S_{i}=2$	0.058
		(−1.793, −1.684)		(0.047, 0.067)
3 [high intercept, low slope]	$\tau_{0}|S_{i}=3$	−0.775	$\tau_{1}|S_{i}=3$	0.022
		(−0.879, −0.681)		(0.008, 0.037)

**Table 4:** Characteristics and original treatment assignment of subjects in the weight loss management study by location LCs determined by posterior classifying probabilities

Characteristic	Location LC 1	Location LC 2	Location LC 3

	[low intercept. most negative slope]	[mid intercept, mid slope]	[high intercept, least negative slope]

	(N=132)	(N=179)	(N=68)

Biological gender			
Female	114 (86.4%)	134 (74.9%)	40 (58.8%)
Male	18 (13.6%)	41 (22.9%)	28 (41.2%)
Age at screening (years)			
Mean (SD)	40.6 (11.9)	40.0 (10.9)	39.1 (10.5)
Ethnicity			
Hispanic or Latino	21 (15.9%)	17 (9.5%)	7 (10.3%)
Not Hispanic or Latino	110 (83.3%)	160 (89.4%)	59 (86.8%)
Race			
White	81 (61.4%)	129 (72.1%)	54 (79.4%)
Black or African American	34 (25.8%)	34 (19.0%)	5 (7.4%)
Others	12 (9.1%)	12 (6.7%)	5 (7.4%)
Weight at baseline (lbs)			
Mean (SD)	203.5 (35.5)	213.8 (33.6)	227.7 (32.8)
Height at baseline (in)			
Mean (SD)	64.6 (6.5)	66.1 (3.2)	68.0 (3.3)
Highest level of education at baseline			
High school graduate/Some college/Technical school	23 (17.4%)	19 (10.6%)	5 (7.4%)
College graduate	109 (82.6%)	160 (89.4%)	63 (92.6%)
Employment status at baseline			
Employed	113 (85.6%)	165 (92.2%)	58 (85.3%)
Unemployed/Homemaker/Student/Retired	19 (14.4%)	14 (7.8%)	10 (14.7%)
Relationship status at baseline			
Married/living with partner	63 (47.7%)	98 (54.7%)	44 (64.7%)
Divorced/Widowed/Separated/Never married	69 (52.3%)	81 (45.3%)	24 (35.3%)
Original treatment assignment			
mHealth only	69 (52.3%)	80 (44.7%)	35 (51.5%)
mHealth + coaching	63 (47.7%)	99 (55.3%)	33 (48.5%)

Observations with missing values for each respective variable are excluded

**Table 5:** Characteristics and original treatment assignment of subjects in the weight loss management study by scale LCs determined by posterior classifying probabilities

Characteristic	Scale LC 1	Scale LC 2	Scale LC 3

	[low intercept, mid slope]	[mid intercept, high slope]	[high intercept, low slope]

	(N=78)	(N=200)	(N=101)

Biological gender			
Female	56 (71.8%)	159 (79.5%)	73 (72.3%)
Male	21 (26.9%)	40 (20.0%)	26 (25.7%)
Age at screening (years)			
Mean (SD)	43.1 (10.8)	39.3 (10.8)	39.0 (11.8)
Ethnicity			
Hispanic or Latino	4 (5.1%)	26 (13.0%)	15 (14.9%)
Not Hispanic or Latino	73 (93.6%)	171 (85.5%)	85 (84.2%)
Race			
White	61 (78.2%)	138 (69.0%)	65 (64.4%)
Black or African American	9 (11.5%)	38 (19.0%)	26 (25.7%)
Others	7 (9.0%)	18 (9.0%)	4 (4.0%)
Weight at baseline (lbs)			
Mean (SD)	204.1 (32.9)	211.7 (35.1)	221.9 (34.9)
Height at baseline (in)			
Mean (SD)	65.9 (3.7)	65.7 (5.7)	66.3 (3.4)
Highest level of education at baseline			
High school graduate/Some college/Technical school	7 (9.0%)	19 (9.5%)	21 (20.8%)
College graduate	71 (91.0%)	181 (90.5%)	80 (79.2%)
Employment status at baseline			
Employed	69 (88.5%)	183 (91.5%)	84 (83.2%)
Unemployed/Homemaker/Student/Retired	9 (11.5%)	17 (8.5%)	17 (16.8%)
Relationship status at baseline			
Married/living with partner	51 (65.4%)	103 (51.5%)	51 (50.5%)
Divorced/Widowed/Separated/Never married	27 (34.6%)	97 (48.5%)	50 (49.5%)
Original treatment assignment			
mHealth only	31 (39.7%)	97 (48.5%)	56 (55.4%)
mHealth + coaching	47 (60.3%)	103 (51.5%)	45 (44.6%)

Observations with missing values for each respective variable are excluded

**Table 6:** Comparison of results from 100 simulations of location-scale regressions with LCs

Parameter	True value	(1) 3 location LCs + 3 scale LCs			(2) 3 location LCs + 1 scale LC			(3) 3 location LCs + no scale component		
		
		Bias	AIW	Coverage (%)	Bias	AIW	Coverage (%)	Bias	AIW	Coverage (%)

$\beta_{0}|L_{i}=1$	6.92	4.88e−04	0.031	95.7	1.04e−04	0.038	97	5.94e−04	0.040	96.9
$\beta_{0}|L_{i}=2$	7.15	−4.20e−04	0.031	93.5	−5.11e−04	0.038	93	4.31e−05	0.040	90.6
$\beta_{0}|L_{i}=3$	7.38	7.46e−04	0.031	97.8	1.54e−03	0.038	99	8.59e−04	0.041	99.0
$\beta_{1}|L_{i}=1$	−0.02	−4.38e−05	0.005	93.5	−2.08e−05	0.006	97	−7.01e−05	0.006	96.9
$\beta_{1}|L_{i}=2$	−0.01	7.52e−05	0.005	94.6	−4.56e−05	0.006	96	−1.21e−04	0.006	97.9
$\beta_{1}|L_{i}=3$	−0.01	−4.56e−05	0.005	98.9	4.87e−05	0.006	97	8.05e−05	0.006	95.8
$\tau_{0}|S_{i}=1$	−2.41	1.05e−03	0.103	94.6	0.98	0.060	0	–	–	–
$\tau_{0}|S_{i}=2$	−1.74	2.18e−03	0.106	93.5	0.31	–	0	–	–	–
$\tau_{0}|S_{i}=3$	−0.78	5.23e−03	0.107	94.6	−0.65	–	0	–	–	–
$\tau_{1}|S_{i}=1$	0.02	1.44e−04	0.016	97.8	0.01	0.009	0	–	–	–
$\tau_{1}|S_{i}=2$	0.06	−7.34e−05	0.016	98.9	−0.03	–	0	–	–	–
$\tau_{1}|S_{i}=3$	0.02	4.93e−05	0.016	95.6	0.01	–	0	–	–	–

*AIW* average 95% credible interval width

## Data Availability

SMART data can be made available by sending a manuscript proposal and a data use agreement to Bonnie Spring, bonnie.spring@med.fsu.edu.

## Code availability

Code is included in the Supporting Information.

## References

[R1] Bielak AA, Cherbuin N, Bunce D, & Anstey KJ (2014). Intraindividual variability is a fundamental phenomenon of aging: Evidence from an 8-year longitudinal study across young, middle, and older adulthood. Developmental Psychology, 50(1), 143. 23586940 10.1037/a0032650

[R2] Clark SL, & Muthén B (2009). Relating latent class analysis results to variables not included in the analysis.

[R3] Connor S (2020). Underreporting of dietary intake: Key issues for weight management clinicians. Current Cardiovascular Risk Reports, 14, 1–10.

[R4] Dunton GF, Atienza AA, Huh J, Castro C, Hedeker D, & King AC (2013). Applying mixed-effects location scale modeling to examine within-person variability in physical activity self-efficacy. International Journal of Statistics in Medical Research, 2(2), 117–122.

[R5] Elliott MR (2007). Identifying latent clusters of variability in longitudinal data. Biostatistics, 8(4), 756–771. 17267391 10.1093/biostatistics/kxm003

[R6] Hedeker D, Mermelstein RJ, & Demirtas H (2008). An application of a mixed-effects location scale model for analysis of ecological momentary assessment (EMA) data. Biometrics, 64(2), 627–634. 17970819 10.1111/j.1541-0420.2007.00924.x PMC2424261

[R7] Jiang B, Elliott MR, Sammel MD, & Wang N (2015). Joint modeling of cross-sectional health outcomes and longitudinal predictors via mixtures of means and variances. Biometrics, 71(2), 487–497. 25652674 10.1111/biom.12284 PMC4480207

[R8] Jones BL, Nagin DS, & Roeder K (2001). A sas procedure based on mixture models for estimating developmental trajectories. Sociological Methods & Research, 29(3), 374–393.

[R9] Lee S-Y, & Song X-Y (2004). Evaluation of the Bayesian and maximum likelihood approaches in analyzing structural equation models with small sample sizes. Multivariate Behavioral Research, 39(4), 653–686. 26745462 10.1207/s15327906mbr3904_4

[R10] Lin X, Mermelstein R, & Hedeker D (2020). Mixed location scale hidden Markov model for the analysis of intensive longitudinal data. Health Services and Outcomes Research Methodology, 20, 222–236.

[R11] Macdiarmid J, & Blundell J (1998). Assessing dietary intake: Who, what and why of under-reporting. Nutrition Research Reviews, 11(2), 231–253. 19094249 10.1079/NRR19980017

[R12] McCulloch CE (2003). Generalized linear mixed models. Institute of Mathematical Statistics.

[R13] Miquelon P, & Castonguay A (2017). Integrated regulation, behavior consistency, and physical activity maintenance. Motivation Science, 3(1), 76.

[R14] Muthén B, & Muthén L (2017). Mplus. van der Linden WJ (Ed.), Handbook of item response theory (1st ed., pp. 507–518). New York, NY: Chapman and Hall/CRC.

[R15] Muthén B, & Shedden K (1999). Finite mixture modeling with mixture outcomes using the EM algorithm. Biometrics, 55(2), 463–469. 11318201 10.1111/j.0006-341x.1999.00463.x

[R16] Nagin DS, & Land KC (1993). Age, criminal careers, and population heterogeneity: Specification and estimation of a nonparametric, mixed Poisson model. Criminology, 31(3), 327–362.

[R17] Nordgren R, Hedeker D, Dunton G, & Yang C-H (2020). Extending the mixed-effects model to consider within-subject variance for ecological momentary assessment data. Statistics in Medicine, 39(5), 577–590. 31846119 10.1002/sim.8429

[R18] Paananen T, Piironen J, Bürkner P-C, & Vehtari A (2021). Implicitly adaptive importance sampling. Statistics and Computing, 31(2), 16.

[R19] Piasecki TM, Hedeker D, Dierker LC, & Mermelstein RJ (2016). Progression of nicotine dependence, mood level, and mood variability in adolescent smokers. Psychology of Addictive Behaviors, 30(4), 484. 26974687 10.1037/adb0000165 PMC4914434

[R20] Picca A, Pesce V, & Lezza AMS (2017). Does eating less make you live longer and better? An update on calorie restriction. Clinical interventions in aging (pp. 1887–1902). 29184395 10.2147/CIA.S126458 PMC5685139

[R21] Redner RA, & Walker HF (1984). Mixture densities, maximum likelihood and the em algorithm. SIAM Review, 26(2), 195–239.

[R22] Rosenbaum DL, Schumacher LM, Schaumberg K, Piers AD, Gaspar ME, Lowe MR, & Butryn ML (2016). Energy intake highs and lows: How much does consistency matter in weight control? Clinical Obesity, 6(3), 193–201. 27020845 10.1111/cob.12142 PMC4864054

[R23] Spring B, Pfammatter A, Scanlan L, Daly E, Reading J, Battalio S, & Nahum-Shani I (2024). An adaptive behavioral intervention for weight loss management: A noninferiority randomized clinical trial. JAMA10.1001/jama.2024.0821 PMC11094642 38744428

[R24] Stan Development Team. (2022). Stan modeling language users guide and reference manual, version 2.31. http://mc-stan.org/

[R25] Tong X, Kim S, & Ke Z (2021). Impact of likelihoods on class enumeration in Bayesian growth mixture modeling. The annual meeting of the psychometric society (pp. 111–120).

[R26] Van Lissa CJ, Garnier-Villarreal M, & Anadria D (2024). Recommended practices in latent class analysis using the open-source r-package tidysem. Structural Equation Modeling: A Multidisciplinary Journal, 31(3), 526–534.

[R27] Vehtari A, Gelman A, & Gabry J (2017). Practical Bayesian model evaluation using leave-one-out cross-validation and waic. Statistics and Computing, 27, 1413–1432.

[R28] Vehtari A, Gelman A, Simpson D, Carpenter B, & Bürkner P-C (2021). Rank-normalization, folding, and localization: An improved *R*ˆ for assessing convergence of mcmc (with discussion). Bayesian Analysis, 16(2), 667–718.

[R29] Verbeke G, & Lesaffre E (1996). A linear mixed-effects model with heterogeneity in the random-effects population. Journal of the American Statistical Association, 91(433), 217–221.

[R30] Weller BE, Bowen NK, & Faubert SJ (2020). Latent class analysis: A guide to best practice. Journal of Black Psychology, 46(4), 287–311.

[R31] Xu W (1995). Mixtures in random-effects regression models (Unpublished doctoral dissertation). University of Illinois at Chicago.

