# Supplementary material for: A latent class location-scale regression model with an application to calorie intake data
Source: J Behav Med. 2026 Jan 13;49(2):275–85. doi: 10.1007/s10865-025-00613-7 (PMC13224735; doi:10.1007/s10865-025-00613-7)
Supplement: Supplementary file 1 — Supplementary file1 (PDF 1541 kb) [file 10865_2025_613_MOESM1_ESM.pdf]

Supporting Information

1 Trace plots for simulation scenarios 2 and 3

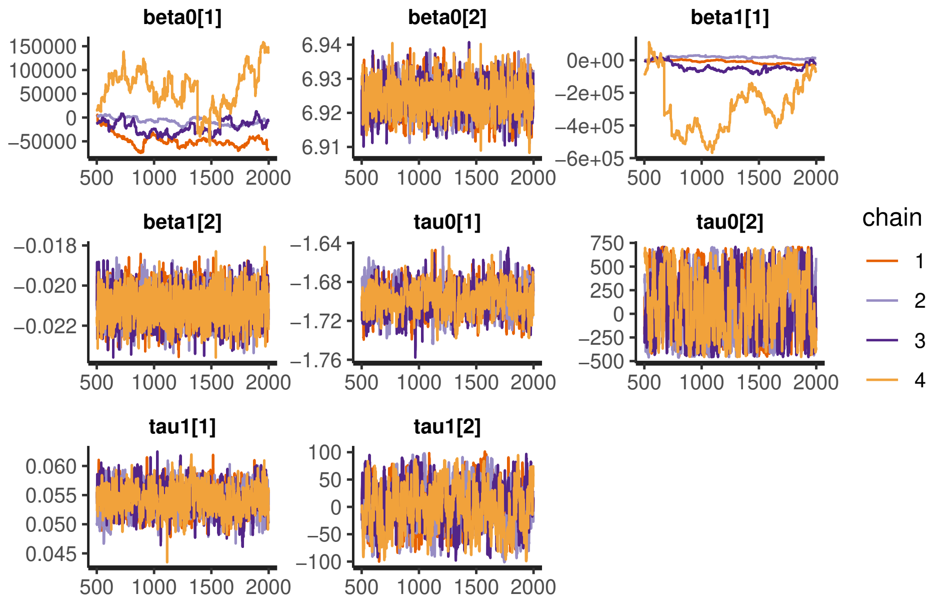

(a) 2 location LCs and 2 scale LCs on data with no LC.

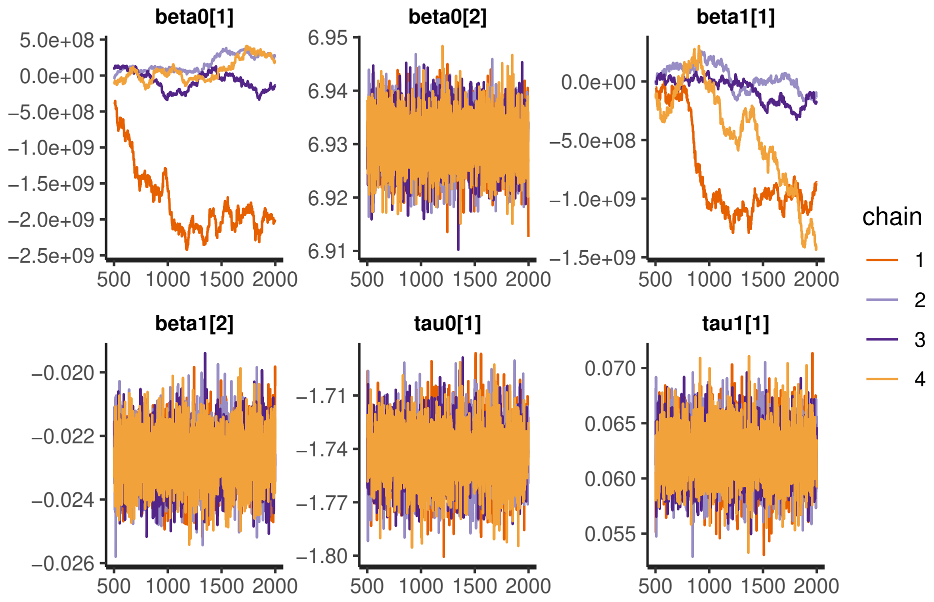

(b) 2 location LCs and 1 scale LC on data with no LC.

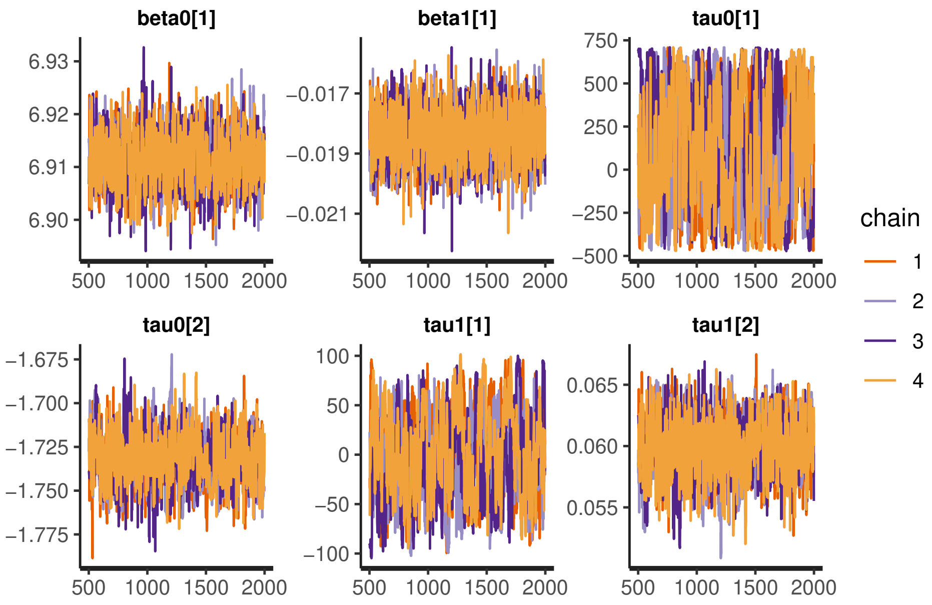

(c) 1 location LC and 2 scale LCs on data with no LC.

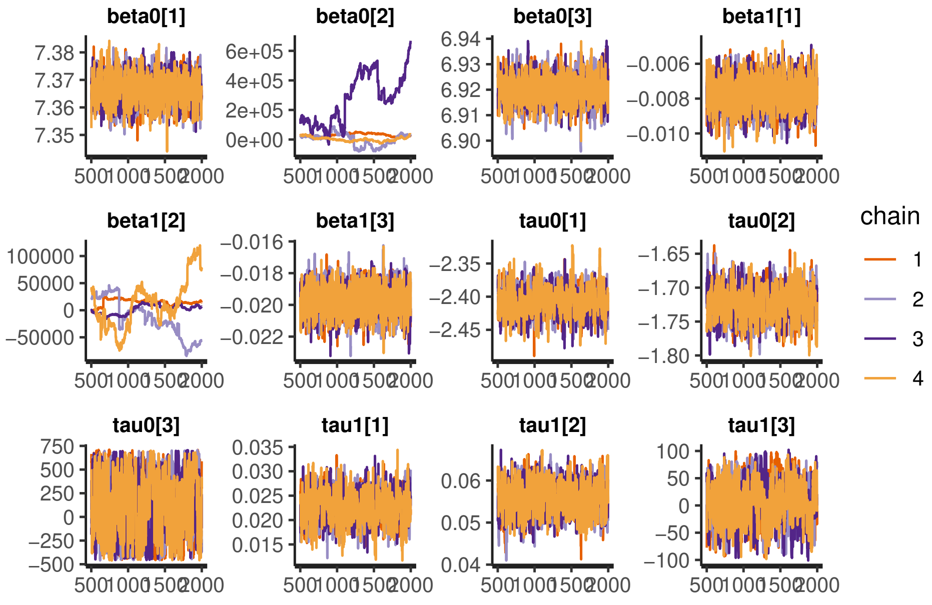

(d) 3 location LCs and 3 scale LCs on data with 2 location and 2 scale LCs.

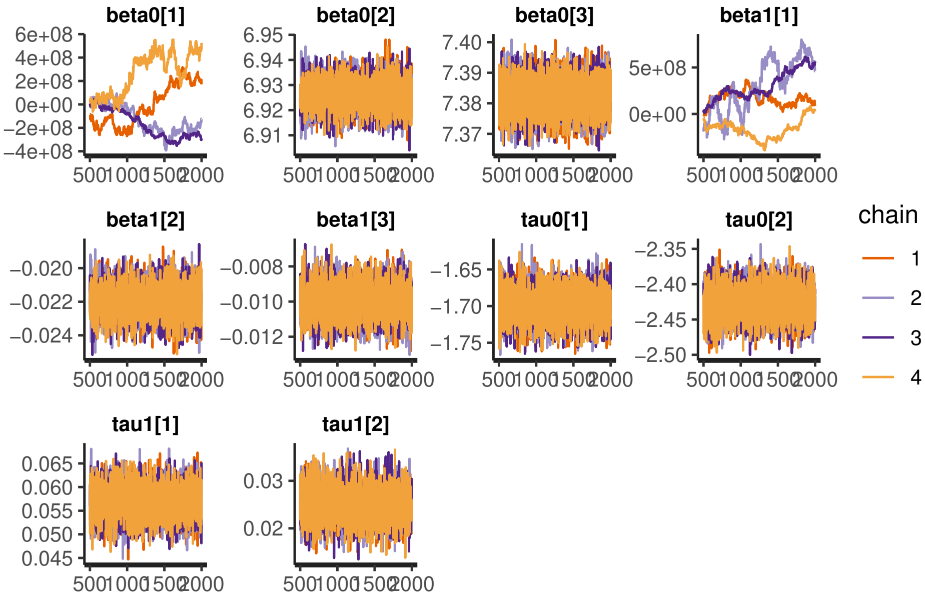

(e) 3 location LCs and 2 scale LCs on data with 2 location and 2 scale LCs.

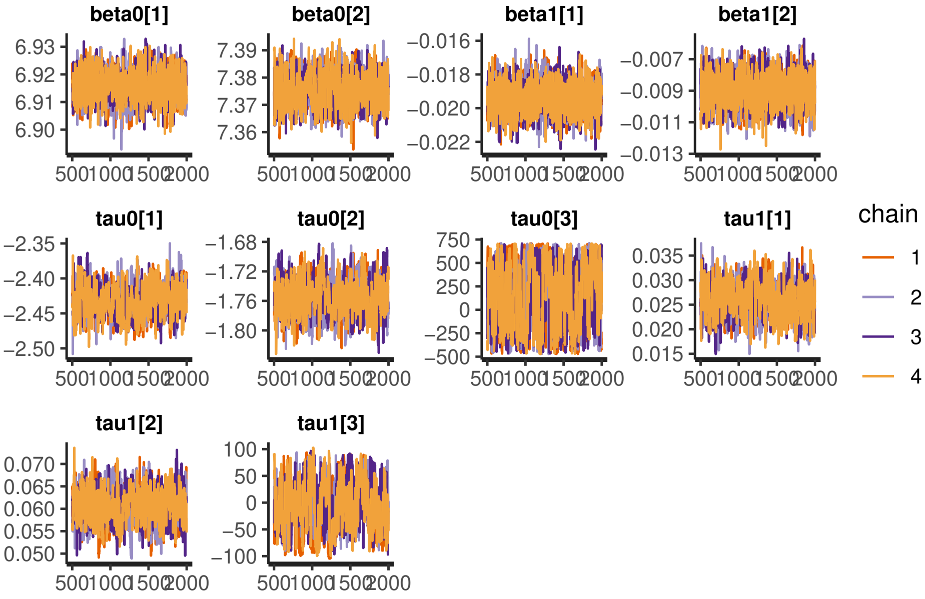

(f) 2 location LCs and 3 scale LCs on data with 2 location and 2 scale LCs.

Figure 1: Trace plots for models with more latent classes than present in the data. For each trace plot in every subfigure, the x-axis represents the number of iterations, and the y-axis represents the parameter estimate.

2 Stan code for model estimation

```
data {
  int<lower=1> K_loc;
  int<lower=1> K_scale;
  int<lower=1> K_total;
  int<lower=1> N; //total number of observations
  int<lower=1> nsubj; //number of subjects
  int<lower=0> n[nsubj + 1];
```

```
  //cumulative sums of observations per subject, with a 0 at beginning for counting purposes
  int<lower=1, upper = nsubj> subject[N];
  int<lower=0> time[N];
  real<lower=0> y[N];
}

parameters {
  //location
  vector[K_loc] beta0;
  vector[K_loc] beta1;

  //scale
  vector[K_scale] tau0;
  vector[K_scale] tau1;

  //mixing proportions
  simplex[K_loc] pi_class_location;
  simplex[K_scale] pi_class_scale;
}

model{
  vector[K_loc] log_pi_location = log(pi_class_location);
  vector[K_scale] log_pi_scale = log(pi_class_scale);//cache log calculation
  vector[K_total] log_pi;

  //likelihood
  for (j in 1:nsubj){
    vector[K_total] lps = rep_vector(0, K_total);

    for(i in (n[j]+1):n[j + 1]){
      for(k_loc in 1:K_loc){
        for(k_scale in 1:K_scale){
          log_pi[(k_loc - 1)*K_scale + k_scale] = log_pi_location[k_loc] + log_pi_scale[k_scale];

          lps[(k_loc - 1)*K_scale + k_scale] +=  normal_lpdf(y[i] |
          beta0[k_loc] + beta1[k_loc] * time[i],
          sqrt(exp(tau0[k_scale] + tau1[k_scale] * time[i])));
        }
      }
    }

    target += log_sum_exp(log_pi + lps);
  }
}

generated quantities{
  vector[nsubj] log_lik;
  vector[K_loc] log_pi_location = log(pi_class_location);
  vector[K_scale] log_pi_scale = log(pi_class_scale);//cache log calculation
  vector[K_total] log_pi;
  vector[K_loc] pi_class_location_subj[nsubj];
  vector[K_scale] pi_class_scale_subj[nsubj];
  //posterior classifying probabilities for each subject
  matrix[K_scale, K_loc] scale_pi;
  matrix[K_scale, K_loc] scale_lps;
  for(k_loc in 1:K_loc){
    for(k_scale in 1:K_scale){
      log_pi[(k_loc - 1)*K_scale + k_scale] = log_pi_location[k_loc] + log_pi_scale[k_scale];
```

```
      scale_pi[k_scale, k_loc] = log_pi_location[k_loc] + log_pi_scale[k_scale];
    }
  }

  for (j in 1:nsubj){
    vector[K_total] lps = rep_vector(0, K_total);

    for(k_loc in 1:K_loc){
      for(k_scale in 1:K_scale){
      scale_lps[k_scale, k_loc] = 0;
      }
    }

    for(i in (n[j]+1):n[j + 1]){
      for(k_loc in 1:K_loc){
        for(k_scale in 1:K_scale){
          scale_lps[k_scale, k_loc] += normal_lpdf(y[i] |
          beta0[k_loc] + beta1[k_loc] * time[i],
          sqrt(exp(tau0[k_scale] + tau1[k_scale] * time[i])));

          lps[(k_loc - 1)*K_scale + k_scale] +=  normal_lpdf(y[i] |
          beta0[k_loc] + beta1[k_loc] * time[i],
          sqrt(exp(tau0[k_scale] + tau1[k_scale] * time[i])));
        }
      }
    }

    log_lik[j] = log_sum_exp(log_pi + lps);

    for(k_loc in 1:K_loc){
      pi_class_location_subj[j,k_loc] = exp(log_sum_exp(log_pi[((k_loc -1) * K_scale + 1):
      (k_loc * K_scale)] +
      lps[((k_loc -1) * K_scale + 1):(k_loc * K_scale)]) - log_lik[j]);
    }

    for(k_scale in 1:K_scale){
      pi_class_scale_subj[j,k_scale] = exp(log_sum_exp(scale_pi[k_scale, 1:K_loc] +
      scale_lps[k_scale, 1:K_loc]) - log_lik[j]);
    }

  }
}
```

3 R code for simulated data generation

```
DataGen <- function(nsubj = 250, ntime = 12, nobs = 10,
                    prob_loc = rep(1/3, 3),
                    prob_scale = rep(1/3, 3),
                    Cloc = 3,
                    Cscale = 3,
                    beta0 = NULL,
                    beta1 = NULL,
                    tau0 = NULL,
                    tau1 = NULL){

  N <- nsubj * ntime * nobs
  subject <- rep(1:nsubj, each = ntime * nobs)
  time <- rep(rep(0:(ntime - 1), each = nobs), nsubj)

  # Class membership
  c_loc <- rep(sample(1:Cloc, size = nsubj, replace = TRUE, prob = prob_loc),
  each = ntime * nobs)
  c_scale <- rep(sample(1:Cscale, size = nsubj, replace = TRUE, prob = prob_scale),
  each = ntime * nobs)

  y <- rep(NA, N)

  for(i in 1:N){
    y[i] <- rnorm(1, mean = beta0[c_loc[i]] + beta1[c_loc[i]] * time[i],
    sd = sqrt(exp(tau0[c_scale[i]] + tau1[c_scale[i]] * time[i])))
  }

  df.LC <- data.frame("subject" = subject, "time" = time,
  "c_loc" = c_loc, "c_scale" = c_scale, "y" = y)

  return(df.LC)
}
```
